# Medical Dispute Committees in the Netherlands: a qualitative study of patient expectations and experiences

**DOI:** 10.1186/s12913-022-08021-2

**Published:** 2022-05-16

**Authors:** Rachel I. Dijkstra, Nieke A. Elbers, Roland D. Friele, Antony Pemberton

**Affiliations:** 1 Tilburg Law School, Tilburg University, PO Box 90153, 5000 LE Tilburg, the Netherlands; 2 Netherlands Institute for the Study of Crime and Law Enforcement, PO Box 71304, 1008 BH Amsterdam, the Netherlands; 3 VU University Amsterdam, De Boelelaan 1105, 1081 HV Amsterdam, the Netherlands; 4 Netherlands Institute for Health Services Research (NIVEL), PO Box 1568, 3500 BN Utrecht, the Netherlands; 5 Tranzo Scientific Center for Care and Wellbeing, Tilburg University, PO Box 90153, 5000 LE Tilburg, the Netherlands; 6 Leuven Institute of Criminology, KU Leuven, Herbert Hooverplein 9, 3000 Louvain, Belgium

**Keywords:** Medical dispute committees, Health care incident, Patients, Complaint, Complaint procedure, Epistemic injustice, Being heard

## Abstract

**Background:**

Health care incidents, such as medical errors, cause tragedies all over the world. Recent legislation in the Netherlands has established medical dispute committees to provide for an appeals procedure offering an alternative to civil litigation and to meet the needs of clients. Dispute committees incorporate a hybrid procedure where one can file a complaint and a claim for damages resulting in a verdict without going to court. The procedure is at the crossroads of complaints law and civil litigation. This study seeks to analyze to what extent patients and family members’ expectations and experiences with dispute committees match the goals of the new legislation.

**Methods:**

This qualitative, retrospective research includes in-depth, semi-structured, face-to-face interviews with patients or family members who filed a complaint with a dispute committee in the Netherlands. The researchers conducted an inductive, thematic analysis of the qualitative data.

**Results:**

A total of 26 interviews were held with 30 patients and family members. The results showed that participants particularly felt the need to be heard and to make a positive impact on health care. Some wished to be financially compensated, for others money was the last thing on their mind. The results demonstrated the existence of unequal power relationships between participants and both the defendant and dispute committee members. Participants reported the added value of (legal) support and expressed the need for dialogue at the hearing. Participants sometimes experienced closure after the proceedings, but often did not feel heard or felt a lack of a practical outcome and a tangible improvement.

**Conclusions:**

This study shows that participants’ expectations and experiences were not always met by the current set up of the dispute committee proceedings. Participants did not feel heard, while they did value the potential for monetary compensation. In addition, some participants did not experience an empowered position but rather a feeling of a power misbalance. The feeling of a power misbalance and not being heard might be explained by existing epistemic injustice, which is a concept that should be carefully considered in processes after health care incidents.

**Supplementary Information:**

The online version contains supplementary material available at 10.1186/s12913-022-08021-2.

## Background

Health care incidents, such as medical errors [1], cause tragedies all over the world. Patients, family members, health care professionals, and even health care institutions suffer the consequences. A recent prospective qualitative study shows problems for victims of health care incidents in terms of “mental health, work, religion, finances and legal issues” [2]. Procedures that try to provide answers to health care incidents include formal (legal) processes, such as medical malpractice litigation. There are also more informal or institutional procedures, such as open disclosure policies and programs, communication-and-resolution programs (in the USA), and complaints procedures, which could be alternatives for the more costly and time-consuming civil litigation [3, 4]. In the Netherlands new institutions, namely dispute committees, were made mandatory by the Dutch Quality, Complaints, and Disputes in Health Care Act (‘Wet Kwaliteit Klachten en Geschillen Zorg’ or Wkkgz) in 2016.

Dispute committees incorporate a hybrid procedure where one can both file a complaint and a claim for damages after a health care incident and receive a verdict without going to court. This procedure is at the crossroads of complaints law and civil litigation. If a client’s complaint cannot be solved internally at the health care institution – which is the preferred option in the Dutch system [5, 6] – dispute committees potentially offer them two elements. First, dispute committees can serve as an independent appeals procedure to having your complaint heard and can administer a binding and final verdict. Second, dispute committees provide an accessible and affordable alternative to civil litigation [6] and can grant damage claims up to €25.000 (article 20 Wkkgz).

The combination of a complaint and a claim at dispute committees is meant to stay within the realm of complaints law, which is there to strengthen the complainants’ position, instead of civil law and its adversarial liability [7]. The government intended a more “integral” or aggregated approach towards claims and complaints with the new legislation, a “culture shift”, to better address the client’s needs and expectations [6, 7]. Civil litigation has been shown to be slow, (very) expensive, and not necessarily aiding the best interests of all parties involved [8, 9]. The new legislation aims for health care and policy to reason from the client’s perspective and strengthen their position [5, 10], to provide for a less legal approach [6] and thereby potentially be less "damaging" and more equipped to restore the relationship between complainant and defendant [6]. Furthermore, dispute committees are meant to offer a just procedure, including elements of a fair hearing, such as hearing both sides of the dispute [11]. If serving properly, dispute committees could strengthen a feeling of justice on the side of clients and improve trust that filing a complaint is meaningful [6].

All health care providers in the Netherlands are obliged to be affiliated with one of the dispute committees (article 18 Wkkgz) since January 1, 2017 [12]. The number of dispute committees has grown from a total of five in 2015 [13] to 41 in 2022 focusing on most types of health care, for example hospital care and midwifery. Dispute committees in 2019 received 627 complaints and 424 were finalized at the time that the study was conducted [14]. Of these complaints 23 were well-founded, 114 partially well-founded, and 165 unfounded. In 102 cases the dispute was resolved outside of the dispute committee through alternative resolution or settlements. Most complaints were related to hospital care, care by general practitioners, and mental health care [14]. Financial claims that are granted generally range between 50 to 5,400 euro, though the information in annual reports is limited [14].

Patients and family members can file a written account of their complaint (article 21 Wkkgz) with the dispute committee through an online system, for which they pay a fee (usually between 50–150 euro) [12], after which a hearing is scheduled. Clients are not obliged to have legal representation, to be present at the hearing or to file an additional claim for damages but they are free to do so. Each dispute committee has its own set of guidelines that govern their proceedings. Generally, according to the guidelines, a committee is comprised of three or five independent members [15–17]. One member usually has a legal background (the president), one or two members represent the patient perspective (usually proposed by client or patient organizations), and up to three members represent the perspective of the health care provider (usually medical professionals from the relevant medical specialism). Both the complainant and the defendant have the right to bring (legal) counsel and to be heard. The verdict is rendered by a majority vote though sometimes the medical professionals share one vote together. Both complainant and defendant receive the verdict within six months (article 22 Wkkgz). The verdict comprises a binding decision regarding the complaint (unfounded, well-founded, or partially well-founded) and the financial claim, a motivation, and it can include recommendations. All verdicts are published in an anonymized version. One dispute committee provides for a compliance guarantee regulation in case the defendant does not comply with the verdict. Meaning that either the industry organization guarantees that the defendant honors the verdict – if the health care provider is a member – or the dispute committee, though the health care provider needs to cooperate.

Little is known about how patients and family members experience this new type of conflict resolution after a health care incident [14] and whether those experiences match the intended goals of the new legislation to offer a hybrid procedure at the intersection of complaints law and adversarial civil litigation. Therefore, the current study investigates to what extent patients and family members’ expectations and experiences match the goals of the legislator, it articulates some careful implications, and it offers a potential explanatory factor for some of the results: epistemic injustice. The dispute committees have been officially operational since January 1, 2017, which allowed for this first reflection on their impact.

## Methods

### Aim, design and setting

This research has a qualitative design with a retrospective focus and is part of a larger study to monitor the impact of the new legislation [12]. This research takes the perspective of patients and family members given that both can file a complaint with the dispute committee. The study aims to analyze to what extent patients and family members’ expectations and experiences match the goals of the legislator. The research consists of in-depth interviews with patients and family members who filed a complaint with a dispute committee in the Netherlands.

### Participants

Of the (then operational) 38 dispute committees in the Netherlands, a total of eight were approached to participate in the study, based on their number of cases. Three dispute committees participated: the Dutch Foundation for Consumer Complaints Board (‘De Geschillencommissie Zorg’, including dispute committees in 17 different health care sectors), the Foundation Complaints and Disputes for Primary Care (‘Stichting Klachten en Geschillen Eerstelijnszorg’), and the Foundation Dispute Committee Oral Care (‘Stichting Geschilleninstantie Mondzorg’). The dispute committees were provided with information letters, which they distributed to potential participants. Privacy regulations, such as the European General Data Protection Legislation, precluded the researchers from directly approaching participants.

An approximate of 400 participants, both complainants and defendants, were approached. Participants were eligible if they were at least 18 years old and had completed a dispute settlement in the recent period before April 1^st^ 2018. Participants were excluded in case of cognitive impairment, dementia, psychotic symptoms, intellectual disabilities, and after the researchers reached data saturation. The selection was based on a convenience sample because researchers could not directly approach participants.

In total at least 69 participants responded and were willing to participate. Nine participants withdrew their participation and one interview was not usable due to background noise. At least 15 other participants were rejected because of cognitive impairment and at least four participants were rejected after the researchers had reached saturation. The researchers eventually conducted 26 interviews with 30 complainants (patients and family members).

### Data collection & analysis

The primary researcher together with two research interns (see Acknowledgements) conducted the interviews in the spring and summer of 2019, which were face-to-face, semi-structured and audio recorded. The three researchers were female, between 20–30 years old, and of Dutch descent. Ethical approval was given by the Tilburg Law School Ethics Review Board.

The researchers used an interview guide and topic list that built on chapter III of the new legislation, the underlying goals of this legislation, and research done on experiences with earlier complaints procedures. The interviews lasted 24–120 min and were transcribed verbatim. Participants were interviewed at home or at their or the researcher’s workplace, and signed informed consent prior to the interview. Four interviews were done by telephone. Each participant received a resume of the interview for validation [18].

One researcher (R.D.) conducted an inductive, data-driven, realist thematic analysis of the interviews following the six phases of analysis of Braun and Clarke [19, 20] and using MaxQDA software. The researcher (R.D.) familiarized herself with the data (phase one) before starting the process of open coding and generating initial codes (phase two), while writing memos on the overall impression of the interviews. While coding the researcher quickly started following the built-up of the interview guide in terms of sequence: experiences in the preparatory stages to filing a complaint, during the hearing by the dispute committee, and regarding experienced outcomes. Codes that emerged quickly were the vulnerability of the patient and goals such as being heard.

The codes and patterns were grouped into overarching themes and sub-themes (phase three). After establishing a preliminary set of themes, the themes were reviewed (phase four), and finalized (phase five). During these phases all authors critically assessed the preliminary code tree and discussed the themes. This article discusses the final selection of relevant themes to the research questions (phase six). An example for identifying final themes is given in Table 1. Throughout the results section quotations are also shown to clarify the process of analysis further. For validity purposes all co-authors read through the interviews and cross-checked them with the themes and sub-themes to see whether all relevant themes had been highlighted for this research.

**Table 1:** The steps of the inductive, thematic analysis exemplified by the main theme “Goal: making a positive impact on health care”

Data extract →	Initial coding (phase two) →	Searching for themes (phase three) →	Reviewing themes (phase four) →	Final theme (phase five)
“Because I wanted it to be looked at more closely” (Participant P)	To have a second look	Objective analysis of the incident	Goal: to improve health care	Making a positive impact on health care
“I wanted the health care institution to be investigated” (Participant A)	Investigating the hospital	Aim to understand what happened and make changes		
“I wanted them to get a serious slap on the wrist” (Participant H1)	Being reprimanded Punishment	Making sure the incident does not go by unnoticed	Goal: to learn from the incident	
“I had rather paid 20,000 euro so that they would say: really, from now on we will do things differently in the medical world.” (Participant S)	Doing things differently after an incident Money is not everything	Learning from the incident		
“What I find most important is that such things do not happen again.” (Participant B)	Important that the incident will not happen again	Prevention	Goal: to prevent the incident from happening to others	

## Results

### Participants’ characteristics

A total of 26 interviews were held, in which 30 participants were interviewed. Of the 30 participants there were 14 patients and 16 family members (together in four cases), and 16 females and 14 males. In terms of age 13 participants were 40–60 years old, 13 participants were older than 60 years, one was between 18–40 years old, and the age of three participants is unknown. Participants filed complaints with dispute committees that ranged between types of health care: a nursing home (1), cardiac care (2), psychiatric care (8), the maternity ward (1), emergency care (1), the lung department (2), plastic surgery (1), hospital care (1), home care (1), the neurology department (1), population screening (1), dentistry (1), orthopedic surgery (2), urology and anesthesiology (1) or urology (1), and a sleep therapy institute (1). 

﻿A claim for damages was filed in 17 out of 26 cases, no claim was filed in seven cases, and it is unknown in two cases. The verdict rendered by the dispute committee was well-founded (three cases), partially well-founded (four cases), unfounded (18 cases), or not admissible due to time lapsed (one case). In two cases (well-founded and partially well-founded) the dispute committee also granted the claim for damages. A full overview of the participants is added to this article as a supplementary file. Following the terminology of the dispute committees, the health care provider is referred to as ‘defendant’﻿. Table 2 provides a brief overview of the main themes and subthemes discussed.

**Table 2:** Overview of themes and sub-themes in results-section

Needs and expectations	Being heard and taken seriously Making a positive impact on the quality of health care Financial compensation
The hearing by the dispute committee	Unequal power relationships Value of support Dialogue at the hearing Impartiality of the dispute committee
Outcomes	Being heard versus not being heard Closure A lack of a practical outcome and tangible improvement The verdict
The value of the interview	

### Needs & expectations

#### Being heard and taken seriously

First, many of the participants mentioned ‘being heard’ and ‘taken seriously’ as an important need or expectation. This need was either stated as a primary goal or followed from participants’ notions that they had received or missed it. Some participants stated that they expected to be heard by the dispute committee and considered it to be the main reason for filing their complaint. For example, participant P recalled “*I wanted to be heard. I wanted that my complaints would be dealt with and that I would not be shoved aside*.” Participant F highlighted the need to be heard as distinct from the need for a favorable verdict.

#### Making a positive impact on the quality of health care

Second, many of the participants brought up the need to make a positive impact on the quality of health care. As participant A pointed out: “*I thought, I will not give up, with the goal to improve the health care for my mother-in-law.*” Many participants voiced the need that health care institutions or professionals should learn from the incident and that the complaint filed with the dispute committee could provide for a correction, sometimes through a slap on the wrist. Others emphasized the need to prevent the occurrence of similar experiences in the future, for example participant B who considered “*But what I find most important is that these kinds of situations do not happen again. I find it disgraceful.*”

#### Financial compensation

Third, at least 17 participants filed a financial claim simultaneously with their complaint (see Supplementary file 1). Most of these participants indicated that financial compensation was important to them. Reasons for this were diverse but were related to costs that were a result of the health care incident, for example revalidation costs (Participant J) or immaterial damages (Participant G). Participants in two cases viewed financial compensation as either the “*ultimate compensation*” (Participant O), meaning the best way to deal with the situation, or a form of punishment because “*if you have to pay money, you feel pain*” (Participant D).

In contrast, there was a substantial number of participants for whom money was the last thing on their mind. Some of these participants had filed a claim for damages but reported that monetary compensation was not a motivation for doing so. Instead, they filed their claim for other reasons, including concerns about the quality of health care or the need to be heard. Participant J mentioned that filing the claim was due to increased resentment because of the response by the health care provider: “[…] *and it came to the point where I thought: ‘But now I am going to get them*’”.

### The hearing by the dispute committee

#### Unequal power relationships

Participants described the setup of the hearing, which was formal with a large table positioning the committee on one side and the complainant and defendant on the other side. An element that pervaded through the accounts by participants was the experience of an unequal power relationship during the hearing.

First, some participants mentioned the experience of an unequal power relationship in relation to the defendant. They felt that a defendant seemed untouchable, came from a different social class, used complicated legal vocabulary, and showed a lack of respect. For example, participant H1 recalled *“… the defendant, that is the same class, they are looked at differently. […] I am a simple soul, which is how they look at you.*” Similarly, participant D considered “*[…] what can you do against a physician? So that feeling has to go by taking patients seriously as well.*” (Participant D).

Second, some participants mentioned that they experienced an unequal power relationship towards the dispute committee. They experienced feelings of having to prove everything or fighting a battle, the dispute committee being smarter, and not being fully recognized as a participant at the hearing. For example, participant R2 considered the attitude of the dispute committee: “*[…] we felt from the start, yes, we were not considered fully fledged participants*.” In addition, participant O felt that the dispute committee considered her less reliable due to her mental health.

Third, some participants indicated that the process of filing a complaint would be too complicated for some people, for example because of the language used and the unwritten rules of conduct. As participant S considered “*you should really have an academic level to be able to get through the process. And not just the procedures and the language […] but also the verbal and non-verbal aspects and how such a world, such an academic beehive, how that world works.*” Sometimes participants felt they lacked knowledge regarding specific aspects of health care, for example treatments.

#### Value of support

With regards to the hearing, participants mentioned how they were accompanied by a support person, for example a friend or relative. In addition, several participants mentioned the potential value of a coach or a lawyer: “*Just someone who says: OK, so how are you going to approach this, what are your next moves?*” (Participant O). Several participants specifically mentioned how they felt that they did not stand a chance against lawyers supporting the defendant. They considered that lawyers were experts and that you would need someone to guide and support you to make sure your arguments were heard and not twisted into something you did not mean. As participant H1 recalled “*because [your] lawyer is of course from the same breed as who else is sitting there. They know what to pay attention to. Me as a layman, I don’t know what I should pay attention to.*” Some other participants consciously refrained from bringing someone to the hearing. Some of them mentioned that they felt capable of just going to the hearing and telling their story.

#### Dialogue at the hearing

Several participants mentioned difficulties with the absence of the original health care professional and wanted him or her to be present at the hearing. An unfamiliar representative was no substitute: “*The one appearing at the hearing was someone I had never seen before. How is that possible, […] how can they send someone that you have […] never met in your entire life?*” (Participant Q). Participants wanted to look the defendant in the eye, to listen to their defense, and to express the own view of the situation (Participant M) or have the opportunity to hold the health care professional accountable (Participant K).

#### Impartiality of the dispute committee

Many participants mentioned that they felt that the dispute committee was prioritizing the defendant during the hearing, that they were even in cahoots with each other, or that the outcome of the process was settled in advance. Participant G emphasized “*I felt as if the verdict was already sitting in the top drawer. The president was not interested in anything.*” In one case the participant reported a feeling of partiality because a person from the internal complaint committee was also a member of the dispute committee (Participant D). Some participants mentioned that the sympathy of the dispute committee was centering on the defendant. Other participants *did* find the dispute committee to be impartial.

### Outcomes

#### Being heard versus not being heard

Many participants reported that they did not feel heard. One participant mentioned the utter lack of attention for the “*human side of the story*” and the feeling that the whole process had “*stalled in a legal framework*” (Participant C2). In another case, participants did not feel heard and even “*silenced*” because they had to summarize their complaint to three main items in only five to ten minutes (Participant H1 and H2). If they had known this, they would have prepared differently, although they did not feel that their situation could be summarized like that. Similarly, participant G reported a lack of room to add elements to the written account of his wife. Participant V specifically mentioned that she felt the president of the dispute committee made a mockery of her preparations and careful documentation. Some participants indicated that they felt they had been heard. For example, participant D felt the dispute committee allowed enough time to tell her story.

#### Closure

Some participants mentioned closure as a result. Participant D mentioned the value of a handshake by the defendant after the hearing, which the participant considered sweet and helped her to accept the outcome of the procedure. Some participants mentioned the potential value of an ‘aftercare’ conversation with the dispute committee to be heard or to receive extra explanation. As participant T considered “*I would have appreciated a phone call […] in which we would have been given the opportunity to talk about how the dispute committee had dealt with our complaint, because I could not find anything about that in the verdict.*”

Other participants highlighted the lack of closure after the procedure was finalized, for example because they felt the dispute committee did not address the most essential element of the case. In addition, some participants considered that an apology after the procedure did not or would not have helped. Yet for others this was precisely the element that was missing. For example, participant J considered “*The only thing that was not finished, was that I would have liked the physician to admit: yes, what I did was wrong.*”

Some participants indicated that they would have wanted dialogue, either at the hearing or beforehand, but felt that the culture had hardened because people tend to focus more on financial claims resulting in a more defensive attitude. Participants mentioned for example that they expected or hoped the dispute committee would have had a more mediating role. Some participants emphasized that a conversation with the health care provider or a round table discussion would have more helpful. Participant J considered that “*if my first complaint had been taken seriously […] there would have been no need for a lawyer, a committee, none of it would have been needed*.”

#### A lack of a practical outcome and tangible improvement

Several participants felt that the dispute committee did not do anything, for example conducted no investigation or omitted to address the core of the problem. For two participants this lack of a practical outcome led to issues regarding the monetary compensation. These participants themselves had to make sure they received the claim for damages that was granted (Participants J, M). Two other participants stated the lack of any tangible improvement after winning, and that recognition alone is not sufficient (Participants A, D). Participant A considered “*It has not changed anything. It does not matter at all whether you win or lose*.”

#### The verdict

Some participants considered the verdict to be timely, clear and understandable: “*just really easy to follow*” (Participant J). Yet others expressed that understanding the verdict was challenging, for example participant Z mentioned “*[…] for me it was a bit abracadabra what is written in the verdict.”*

Some participants were displeased with the verdict because of disappointment, frustration, or a lack of trust in the dispute committee’s expertise. Several participants mentioned that in hindsight, they would not have filed a complaint with the dispute committee or would not do so in the future. Some participants said filing the complaint with the dispute committee had only exacerbated the situation. As participant F recalled “*[…] what happened there, has made the situation a lot worse. A whole lot worse. I felt deeply humiliated.*” In contrast, several participants considered the dispute committee, in principle, a functioning institution with integrity and able to serve the participant’s best interests.

### The value of the interview

A notable finding was the value of the interview itself for participants. Almost all participants thanked the interviewer for listening to their story, for example *“… if you can talk to someone about it, who is interested and who is planning to do something with the findings, that is really worth something.*” (Participant G). Many of them specifically thanked the interviewer by e-mail afterwards considering this conversation had helped them to move on and let go. Some said that being heard by the interviewer was something that they needed, which had now (finally) happened and provided some relief. Some did highlight the emotional toll it took to prepare for the interview and to talk about what had happened again.

## Discussion

### Goals of the new legislation

#### An independent appeals procedure and an alternative for civil litigation

The hybrid procedure at dispute committees is at the crossroads of complaints law and civil litigation: it serves as an independent appeals procedure to hear complaints and provides an alternative to civil litigation. Dispute committees as an independent appeals procedure strongly link to providing a just procedure. In healthcare decision-making providing a just procedure by the health care provider is extra relevant, given that patients tend to develop emotional connections to health care providers [21]. This connection presumably persists when a dispute is brought to a dispute committee. Unfortunately, many participants in the present study considered the dispute committee partial and the verdict settled in advance, although other participants mentioned a positive experience regarding this impartiality. The report of a recent survey underlines the experiences of the first category of participants and shows that only 34% of respondents experienced the dispute committee as impartial [14]. Somehow participants were left with the impression that dispute committees were in cahoots with health care providers.

The dispute committees as an alternative to civil litigation corresponds well with the felt need among many participants for financial compensation. Dispute committees enabled them to file a claim for damages in a more accessible and less legal manner. However, a substantial number of participants also emphasized that money was the last thing on their mind. This links to research done on litigation aims in mediated medical malpractice cases, where most plaintiffs considered that it was not (just) about the money but also about admitting that a mistake was made and making sure it would never happen again [22]. Overall the findings in the present study are similar with regards to the theme “it was not about the money” [22] and having multiple other goals, yet many of the participants did value monetary compensation. In one case (participant J) frustration led to filing the claim, underlining scholarly work that shows that a financial claim can be the result of built-up frustration [23]. In terms of providing an alternative, so far dispute committees have not (yet) taken over civil litigation: annual reports by liability insurers do not show a decrease but rather a stabilization in civil lawsuits in the Netherlands [14]. Furthermore, claims that were granted tended to be relatively low (up to 1500 euro) [14].

#### To address the needs of clients and to offer a fair hearing

The legislation also aims to meet the needs of clients and to offer them a fair hearing at dispute committees. The present study shows that two of the most pressing needs of participants were to be heard and to make a positive impact on health care. Unfortunately, many participants did not feel heard and several participants indicated a lack of a practical outcome and a tangible improvement. This shows an “expectations gap” between expectation and outcome [24].

The need to be heard aligns with previous empirical research. Patients and family members in open disclosure processes in Australia, in reconciliation processes in the context of New-Zealand’s no-fault scheme, and in a communication-and-resolution program in the USA highlighted the need to have an open dialogue, to be able to question explanations, and to be heard [25–27]. Dispute committees indeed aim to let complainants feel heard, but, as Moore and Mello considered, listening is not the same as providing a feeling of ‘being heard’. The latter requires open-ended questions and allowing patients to be leading in what they consider most important during conversations [26]. Participants experienced this when interviewed: they felt they were finally heard by the interviewer. This links to research done on the potential therapeutic effects of research interviews [28].

In addition, the need to make a positive impact on health care aligns with patient expectations of Dutch complaints committees: participants overwhelmingly wanted the committee to recommend the hospital to change things [29]. A recent analysis of surveys regarding Dutch dispute committees also reiterates the felt need to improve the quality of care, but only 9% experienced that this happened [14]. Other studies also show the need among patients to make sure a similar event will not happen again, for example regarding open disclosure in Australia and civil liability in the Netherlands [25–27, 30].

####  A strengthened position for clients

Finally, the legislation aims to strengthen the position for clients and to reason from their perspective. However, some participants described the feeling of an unequal power relationship. These participants felt that they were not treated as fully fledged participants by the dispute committees and experienced an imbalance with the defendant who used difficult vocabulary and seemed to come from a different social class. Given this context, some participants missed (legal) support and considered that not having support would make a case impossible to win. Earlier research shows that the presence of a support person in conversations after a health care incident, either a lawyer or other support person, can indeed be of tremendous help during the process [26].

The previous analysis of the legislative goals and the experiences of participants demonstrate that the current hybrid ambitions of the dispute committees have not necessarily rendered all envisioned results and there appears to be an “expectations gap” [24] on the side of participants. Many participants also did not experience the proceedings as impartial. Notably, some participants experienced inferiority and a lack of support. These findings can be understood in the context of epistemic injustice.

### Epistemic injustice

Epistemic injustice is defined as “a wrong done to someone specifically in their capacity as a knower” [31]. Fricker considers two types of epistemic injustice, testimonial injustice and hermeneutical injustice, which Kidd and Carel have analyzed in the context of health care [32]. Testimonial injustice centers on the credibility of a speaker: he or she is considered a less “reliable giver of information” [32], causing someone’s voice to be worth less [31, 32]. Ill people have a high risk of suffering from such testimonial injustice, given that they are, for example, stereotypically portrayed as weaker in the sense of mental or emotional wellness, especially in cases of mental illness [33]. Hermeneutical injustice results from situations where structural prejudices and a lack of (conceptual) resources cause someone to be disadvantaged regarding how they understand and express their experiences [31, 34]. Both testimonial and hermeneutical injustice can occur when an ill persons’ perception of a situation is ignored, for example because this person does not use the words that would render it relevant for epistemic consideration [33]. In contrast, health care professionals are regarded as “epistemically privileged” [33].

Both testimonial and hermeneutical injustice seem to appear in the current study. Some participants repeatedly highlighted that they felt a power misbalance when filing a complaint and many participants did not feel heard. What they expressed links closely to the feeling that a complainant is not as capable of providing reliable information as a defendant. Some participants felt that their perspective and input was not or less credited: their experiential testimony was worth less than the expert testimony of the health care professionals. The dispute committee and defendant seemed to talk to one another on a different linguistic and social level, and many participants felt less important or taken less seriously. The dispute committee largely came from the same ‘breed’ of educated medical and legal professionals. In addition, several participants had trouble to ‘fit’ their story into the procedural frames of timing or the number of arguments they could discuss. It seems inevitable that complainants have less medical knowledge than their medical opponent, the defendant, which in itself causes a danger of epistemic injustice. However, aside from this misbalance, the defendant is often assisted – or replaced – by a legal professional who brings in legal jargon and know-how. Therefore, the complainant is confronted with complicated language and a social reality that is framed by academic educations and the procedural built-up of dispute committees. This can sometimes sit very far from the social reality of the complainant.

### Implications for policy and practice

This study provides a first glance of participants’ expectations and experiences, which provides the opportunity to formulate modest implications. First, dispute committee members should be aware of the danger of epistemic injustice in their proceedings, potentially leading to feelings of inferiority and partiality. It might be prudent to limit the use of legal and medical jargon and to allow complainants a longer or in any case specific timeframe to state their case, for example 20 min. Committee members might benefit from a training program specifically designed to develop (more) sensitivity to the value of experiences and emotions. Knowledge of such potential epistemic injustice could inform the attitude of people involved in other processes after a health care incident as well, such as open disclosure processes.

Second, constructive recommendations in verdicts and offering a compliance guarantee regulation, such as employed by some dispute committees, could be considered best practice to let complainants be heard and to make a tangible improvement by ensuring that verdicts are acted upon. Such a guarantee could potentially be combined with a feedback moment between the health care institution and the complainant on the potential improvements. Reasoning from this angle, it might be an interesting avenue to consider establishing a sort of health care culture analyst at health care institutions whose main task it would be to make sure internal complaints and verdicts at dispute committees or courts are acted upon and implemented.

Finally, in meeting expectations it might be interesting to consider how health complaints entities in Australia and New Zealand have shaped their proceedings. Health complaints entities provide quick, lawyer-free, and affordable dispute resolution through a myriad of processes that complement (Australia) or basically replace (New Zealand) civil litigation [24, 35]. Differently from dispute committees, health complaints commissions appear to be more flexible in how their processes are shaped, and outcomes generated can range from an explanation (an example of early resolution) to a hearing and an official report including recommendations (an example of an investigation). Such a flexible approach might be better suited to meet expectations, especially with regards to the financial claim that was not always the main driver for participants in this study. Though a clear understanding of expectations and managing unrealistic expectations on the side of patients is key [24]. Future research could consider how dispute committees and health complaints entities interlink and what best practices can be extracted, particularly regarding the value of the financial claim in dispute committee proceedings.

### Strengths and limitations

The strengths of the study include providing a thorough understanding of how patients and family members experienced filing a complaint with dispute committees. The validity of the findings was supported by data saturation and a validity check by all authors. The Dutch context provides for a deeper understanding of this procedure in the Netherlands and this national perspective could inform similar processes in an international setting.

A limitation of the study was the inability to directly approach participants. The study was based on a convenience sample, limiting the potential to generalize findings. For example, the number of unfounded complaints in this study (18 out of 26 cases) was higher than the number of unfounded complaints that Friele et al. reported about complaints filed in 2019 (165 out of 424) [14]. Furthermore, themes and information about the process *leading up* to filing a complaint were not included for analysis because of our focus on participants’ experiences *during* the complaints process. Finally, the outcome (well-founded or unfounded) of each case seems to have impacted participants’ experiences. Given the nuanced experiences of all participants, with both positive and negative elements, it was difficult to actively look at a correlation between a well-founded or unfounded complaint and a positive or negative experience with a dispute committee. However, researchers interpreted six (all female) of the 26 cases as an overall positive experience, of which three were well-founded.

## Conclusions

The new legislation is, among other things, designed to provide a stronger position for clients in health care, to reason from their perspective, and to address the client’s needs and expectations. It aims to establish dispute committees that provide an independent appeals procedure and an affordable alternative to civil litigation. This study shows that participants’ needs and expectations were not always met by the current set up of the proceedings. Many participants did not feel heard, while at the same time many participants did value the potential for monetary compensation. In addition, some participants did not experience an empowered position but rather a feeling of a power misbalance between participant and the defendant at the dispute committee hearing. The feeling of a power misbalance and not being heard might be explained by existing epistemic injustice, which is a concept that should be carefully considered in processes after health care incidents.

## Supplementary Information

**Additional file 1.** Overview of participants.

## Data Availability

The data that support the findings of this study are available from the Netherlands Institute for Health Services Research (NIVEL) but restrictions apply to the availability of these data, which were used under license for the current study, and so are not publicly available. Data are however available from the authors upon reasonable request and with permission of the Netherlands Institute for Health Services Research (NIVEL).

## Abbreviations

Wkkgz: Dutch Quality, Complaints, and Disputes in Health Care Act (‘Wet Kwaliteit Klachten en Geschillen Zorg’ or Wkkgz)
