# Supplementary material for: Medical Dispute Committees in the Netherlands: a qualitative study of patient expectations and experiences
Source: BMC Health Serv Res. 2022 May 16;22:650. doi: 10.1186/s12913-022-08021-2 (PMC9109360; doi:10.1186/s12913-022-08021-2)
Supplement: Supplementary file 1 — Additional file 1. Overview of participants. [file 12913_2022_8021_MOESM1_ESM.docx]

**Supplementary file: Overview of participants**

| **Participant** | **Patient/Family member** | **Gender** | **Age** | **Incident**  *According to complainant* | **Type of health care** | **(Main) complaint** | **Financial claim** | **Verdict:** |
| --- | --- | --- | --- | --- | --- | --- | --- | --- |
| A | Family member | Female | 40-60 | Forgot to administer medication (insuline) | Nursing home | Forgot to administer medication (insuline) | No | Well-founded |
| B | Patient | Female | >60 | Early release from hospital | Cardiac care | Early release from hospital. | Yes | Unfounded |
| C1 and C2 | Family member  Family member | Female  Male | >60 | Suicide (zelfdoding) of husband | Psychiatric care | Refusal to share medical files | No | Barred due to time lapsed |
| D | Patient | Female | 18-40 | Disrespectful treatment and no attention for privacy request. | Maternity ward | Disrespectful treatment, no privacy, and no discussion of treatment. | Yes | Well-founded, no financial compensation |
| E | Patient | Female | 40-60 | Wrong diagnosis regarding breast cancer | Population screening | Wrong diagnosis | Yes | Unfounded |
| F | Family member | Female | 40-60 | Deceased brother | Psychiatric care | Insufficient care of brother and disrespectful treatment (intimidation). | No | Unfounded |
| G | Family member | Male | >60 | Inadequate care after broken bone | Emergency care | No painkillers, inadequate orthopedic care, and insufficient information | Yes | Partially well-founded, claim granted |
| H1 and H2 | Family member  Family member | Female  Male | 40-60 | Insufficient monitoring of care for son | Lung department | Insufficient monitoring of care for son and negative impact of hospital approach. | Yes | Unfounded |
| I | Patient | Female | >60 | Disrespectful treatment and no attention for well-being | Cardiac care | Sharing medical files with new doctors and continuous prescription drugs | No | Unfounded |
| J | Patient | Female | 40-60 | Misdiagnosis resulting in partial amputation | Plastic surgery | Misdiagnosis and disrespectful treatment | Yes | Well-founded, claim granted |
| K | Family member | Male | >60 | For hereditary purposes and historiography | Psychiatric care | Refusal to share medical files | Yes | Unfounded |
| L | Family member | Female | >60 | Inadequate care leading to delirium and eventually death of husband | Hospital care | Inadequate care leading to delirium | No | Partially well-founded |
| M | Family member | Female | >60 | Inadequate care and too late and carelessly (not) administering drugs | Home care | Respecting agreements and administering drugs both carefully and on time. | No | Partially well-founded |
| N | Patient | Male | >60 | Drug administration without proper training, protocol, and carefulness | Lung department | Drug administration without proper training, protocol, and carefulness | No | Unfounded |
| O | Patient | Female | 40-60 | Careless and inadequate therapy | Psychiatric care | Careless and inadequate therapy | Yes | Unfounded |
| P | Patient | Male | Unknown | Wrongfully placed hand-injection with neurological damage (paralysis) | Neurology department | Wrongfully placed injection with neurological damage | Yes | Unfounded |
| Q | Family member | Female | Unknown | Statements made by psychologist in court hearing | Psychiatric care | Wrongful statements made by psychologist in court hearing | Yes | Unfounded |
| R1 and R2 | Patient  Family member | Female  Male | 40-60 | Pain in teeth due to improper treatment | Dentistry | Treatment causing pain for months | Yes | Unfounded |
| S | Family member | Male | Unknown | Operation against wishes of patient and no attention for overall well-being of (very ill) patient | Orthopedic surgery | Operation against wishes of the patient | Yes | Unfounded |
| T | Family member | Male | >60 | Operation 10 days after fracture and no aftercare | Orthopedic surgery | Lack of aftercare and material damages | Yes | Unfounded |
| U | Patient | Male | 40-60 | Misdiagnosis and (im)material damages | Psychiatric care | Misdiagnosis and (im)material damages | Yes | Unfounded |
| V | Patient | Female | 40-60 | Sharing medical files without consent and incorrect information | Psychiatric care | Sharing medical files without consent and incorrect information | Yes | Unfounded |
| W1 and W2 | Patient  Family member | Male  Female | >60 | Pain because of incorrect epidural | Urology/  Anesthesiology | Recognition of incorrect epidural and material damages | Yes | Unfounded |
| X | Patient | Male | 40-60 | Psychiatric treatment for too long and informing GP without consent | Psychiatric care | Retract diagnosis/care plan | Yes | Partially well-founded |
| Y | Family member | Male | >60 | Deceased wife | Urology/General hospital care | Wrongful diagnosis and mistreatment | Unknown | Unfounded |
| Z | Patient | Male | 40-60 | Lack of proper treatment | Sleep therapy institute | Lack of proper treatment | Unknown | Unfounded |
